# Effect of electroacupuncture on cyclic adenosine monophosphate-protein kinase A-vanillic acid receptor subtype 1 of the transient receptor potential/PLK-protein kinase C-vanillic acid receptor subtype 1 of the transient receptor potential pathway based on RNA-seq analysis in prostate tissue in rats with chronic prostatitis/chronic pelvic pain syndrome

**DOI:** 10.3389/fnins.2022.938200

**Published:** 2022-08-24

**Authors:** Xiao-Ling Wu, Kai Cheng, Chang Xu, Ye-Mao Chai, Tai-Heng Yap, Zhi-Wen Yang, Qian-Hui Sun, Yan Tan, Jia-Ni Zhang, Wei Chen, Xing-Hua Qiu, Xing-Yue Yang, Na Li

**Affiliations:** ^1^College of Acupuncture-Moxibustion and Tuina, Beijing University of Chinese Medicine, Beijing, China; ^2^College of Academy of Life Sciences, Beijing University of Chinese Medicine, Beijing, China; ^3^Innovative Institute of Chinese Medicine and Pharmacy, Chengdu University of Traditional Chinese Medicine, Chengdu, China

**Keywords:** chronic prostatitis/chronic pelvic pain syndrome, electroacupuncture, RNA-seq technology, analgesic mechanism, animal experiment

## Abstract

**Objective:**

To investigate the analgesic mechanism of electroacupuncture (EA) in rats with chronic prostatitis/chronic pelvic pain syndrome (CP/CPPS).

**Methods:**

Thirty male SD rats were randomly divided into sham group, model group and EA group, with ten rats in each group. The CP/CPPS model was prepared by injecting 50 μL of complete Freund’s adjuvant (CFA) into the ventral lobes of the prostate tissue, and the sham group was injected with the same dose of saline. After 14 days of modeling, EA was applied to Guanyuan (CV4), Zhongji (CV3), Sanyinjiao (SP6) and Huiyang (BL35) in the EA group. After four courses, H&E staining was performed to observe the prostate tissue morphology, transcriptome sequencing (RNA-Seq) was performed for each group, and the selected signaling pathways were verified by qRT-PCR.

**Results:**

The RNA-Seq analysis results suggested that the analgesic effect of EA on CP/CPPS may be achieved by regulating prostate gene expression, which may be related to multiple biological processes and signaling pathways. qRT-PCR results showed that the vanillic acid receptor subtype 1 of the transient receptor potential (TRPV1), phospholipase C (PLC), protein kinase C (PKC), cyclic adenosine monophosphate (cAMP), and protein kinase A (PKA) were all upregulated in the model group compared to the sham group (*p* < 0.01). Compared with the model group, TRPV1, PLC, PKC, cAMP, and PKA were all downregulated in the EA group (*p* < 0.05, *p* < 0.01).

**Conclusion:**

The analgesic mechanism of EA on CP/CPPS may be achieved through modulation of cAMP-PKA-TRPV1/PLC-PKC-TRPV1 signaling pathway.

## Introduction

Prostatitis is one of the most common urinary diseases in adult men. Based on the National Institutes of Health Prostatitis Collaborative Network classification, chronic prostatitis/chronic pelvic pain syndrome (CP/CPPS) is referred to as category III prostatitis, which is the most common chronic prostatitis, accounting for about 90∼95% of prostatitis (Chen et al., 2021). Its main characteristics are persistent pelvic pain, urinary tract irritation and sexual dysfunction. Among them, pain is the main symptom of most CP/CPPS patients. Long-term and repeated symptom of pain will bring negative psychological effects, substantial health care costs and seriously affect their daily life (McNaughton and Pontari, 2001).

At present, the clinical treatments for CP/CPPS mainly include alpha-blockers, antibiotics and non-steroidal anti-inflammatory drugs (Cohen et al., 2012). However, these drugs’ treatment has obvious negative effects, such as dizziness, nausea, and postural hypotension and gastrointestinal side effects, which reduce patients’ compliance with treatments (Nickel et al., 2013). Despite recent advances in novel treatment schemes development, its clinical effectiveness needs further appraisal (Engeler et al., 2013). Previous studies indicated that EA may be a potential therapy for CP/CPPS (Qin et al., 2016a,b).

EA, a special modern type of acupuncture, originates from the combination of traditional acupuncture and modern electrical stimulation (Cao et al., 2021). With the advantages of inexpensive, safe and fewer side effects, in recent years, EA has been widely used in the prevention and rehabilitation of CP/CPPS (Franco et al., 2018). A multicenter, randomized, sham-controlled trial showed that EA can alleviate the symptoms of pain, micturition dysfunction, anxiety and depression in patients with CP/CPPS, and its efficacy may last for 24 weeks after treatment (Sun et al., 2021). While the efficacy of EA on CP/CPPS has been proven in clinical practice, little is studied and elucidated, however, about the analgesic mechanism. Most animal experiments related to the mechanism of EA on CP/CPPS are without in-depth and systematic exploration of possible signal pathways and targets (Wu et al., 2021).

**FIGURE 1:**
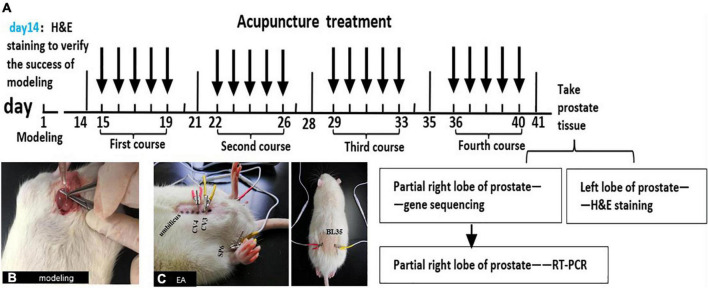
The schematic diagram of this experiment. **(A)** The schematic diagram for methodologies. Male SD rats were injected with CFA into the ventral lobes of the prostate on both sides on the first day, and H&E staining was used to judge whether the model was successful or not on the 14th day. All groups were treated on the 15th day after modeling. Treatment once a day for 40 min, 5 days as a course of treatment, with 2 days of rest between the two courses, a total of 4 courses. At the end of the last course of treatment, the rats were anesthetized and sacrificed on the next day and take the prostates. The left lobe of the prostate was stained with H&E, and partial right lobes were analyzed with RNA-Seq technology. Then, combined with the results of RNA-Seq and modern research progress on the pain-related mechanism of CP/CPPS, the DEG and pain-related signal pathways were screened out. Finally, the key genes in the pathways were verified by qRT-PCR. **(B)** Injecting CFA into the prostate of rats to make CP/CPPS model. **(C)** Acupoint location and EA process in CP/CPPS rats. CV3(zhongji), CV4(guanyuan), SP6(sanyinjiao), and BL35(huiyang).

Transcriptome sequencing (RNA-Seq) is a novel method for gene expression profiling by next-generation sequencing of transcripts (Shi et al., 2018). With the advantages of high sensitivity, high throughput and low cost, it has been widely used to explain disease gene expression signatures. For example, Zhao (2018) used RNA-Seq technology to preliminarily explore the possible mechanism of CP/CPPS. However, studies related to the analgesic mechanism of EA on CP/CPPS using RNA-Seq technology have been not reported. In this study, we tried to comprehensively elucidate the potential molecular mechanisms underlying the analgesic effects of EA on CP/CPPS rats using RNA-Seq technology and provide a scientific basis in clinics (Figure 1).

## Results

### The results of H&E staining of the prostate tissue

**FIGURE 2:**
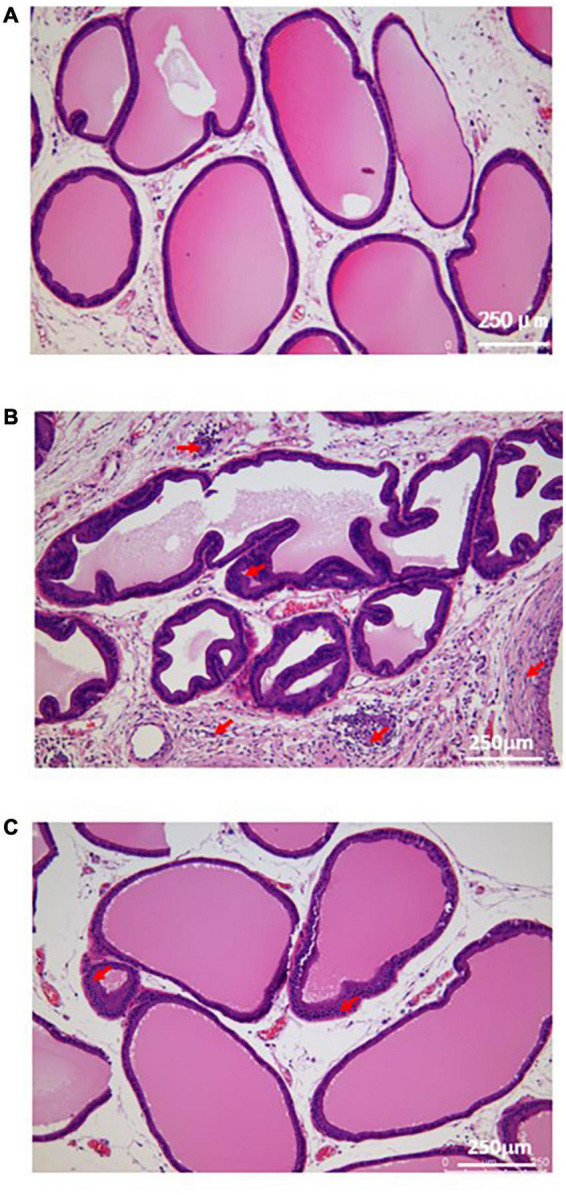
Histological morphology of prostatic tissue in each group. **(A)** The result of H&E staining in sham group; **(B)** The result of H&E staining in model group; **(C)** The result of H&E staining in EA group. Red arrows show the infiltration of inflammatory cells.

In the sham group, the cavity was filled with evenly distributed pink secretion, without inflammatory cell infiltration in the glandular cavity and stroma. In the model group, the prostatic epithelium was papillary hyperplasia, with a narrow glandular cavity, and the pink secretion in the cavity was reduced, with a large number of inflammatory cells in the glandular cavity and stroma. In the EA group, the pathological changes were significantly improved, with the basically complete structure of the glandular cavity, and the inflammatory cell infiltration was rare in the glandular cavity and stroma, with more pink secretions in the cavity (Figure 2).

### Bioinformatics analysis of rat prostate tissue

#### Analysis results of quality control

To ensure the quality of the original sequencing data, it is necessary to evaluate the quality of the original data before analysis. The results showed that 670 million reads were obtained, with an average of about 44.49 million reads per sample; Q20 of all samples ranged from 98.3 to 98.63%, Q30 ranged from 95.13 to 95.71%, and 92.78–96.26% of all samples could map to the reference genome, indicating that the sequencing results were reliable (Table 1).

**TABLE 1:** Quality control and sequencing information for samples.

NO	Sample	Raw reads	Clean reads	Q20 (%)	Q30 (%)	Total mapped
1	Sham1	41,225,988	40,457,396	98.63	95.71	38,413,513 (94.95%)
2	Sham2	42,578,460	40,854,460	98.37	95.23	39,026,425 (95.53%)
3	Sham3	44,344,402	42,449,774	98.33	95.14	40,252,894 (94.82%)
4	Sham4	45,633,734	43,329,820	98.37	95.15	41,165,825 (95.01%)
5	Sham5	44,166,684	41,622,168	98.32	95.19	39,459,030 (94.8%)
6	Mode1	45,693,778	43,839,792	98.3	95.13	41,732,871 (95.19%)
7	Mode2	45,900,210	43,959,880	98.42	95.4	41,801,767 (95.09%)
8	Mode3	47,510,362	45,683,786	98.49	95.46	43,685,288 (95.63%)
9	Mode4	45,538,110	44,545,936	98.41	95.37	42,239,898 (94.82%)
10	Mode5	46,465,170	44,539,906	98.58	95.68	42,775,386 (96.04%)
11	EA1	42,013,976	40,135,674	98.37	95.26	38,633,546 (96.26%)
12	EA2	42,207,288	40,110,152	98.43	95.39	38,229,295 (95.31%)
13	EA3	45,244,586	42,868,484	98.5	95.4	40,910,076 (95.43%)
14	EA4	42,968,022	41,204,250	98.37	95.17	39,316,288 (95.42%)
15	EA5	45,988,586	43,599,800	98.42	95.28	40,449,826 (92.78%)

**(1)** Sample, sample name; 15 cDNA libraries are sham group (sham1, 2, 3, 4, and 5), model group (model1, 2, 3, 4, and 5), EA group (EA1, 2, 3, 4, and 5); **(2)** raw reads, counting the number of original sequence data; **(3)** clean reads, counting the number of sequencing data after filtering; **(4)** Q20, Q30, counting Phred values, respectively. **(5)** Total mapped, the number of clean reads that can be located on the genome.

#### Results of correlation heat map

**FIGURE 3:**
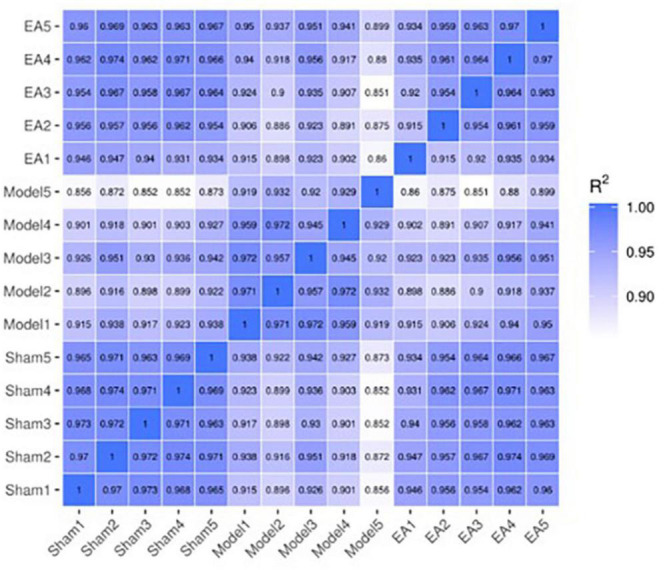
Correlation heat map between samples. The correlation heat map between samples is often used to evaluate the sample difference between groups and the sample repetition within groups. The *X*-axis and *Y*-axis in the above figure are sham group (sham1, 2, 3, 4, and 5), model group (model1, 2, 3, 4, and 5) and EA group (EA1, 2, 3, 4, and 5). The values between the *X*-axis and *Y*-axis are the square of the correlation coefficient between the corresponding samples. The darker the color indicates the higher the correlation coefficient between samples, and the lighter the color indicates the lower the correlation coefficient between samples.

Through the correlation heat map, it could be seen that the correlation coefficient between samples in each group was high, which revealed the high similarities in expression patterns between samples in each group, and further indicated good experiment repeatability, as well as a reasonable selection of samples in each group. The correlation coefficient between the model group and the sham group was relatively low, which revealed that there were some differences in the expression pattern between the model group and the sham group. After EA intervention, compared with the correlation coefficient between the model group and the sham group, the correlation coefficient between the EA group and the sham group was relatively high, which indicated that after EA intervention, the expression pattern of rats in the EA group tended to that of rats in the sham group (Figure 3).

#### Analysis results of differential expression gene

**FIGURE 4:**
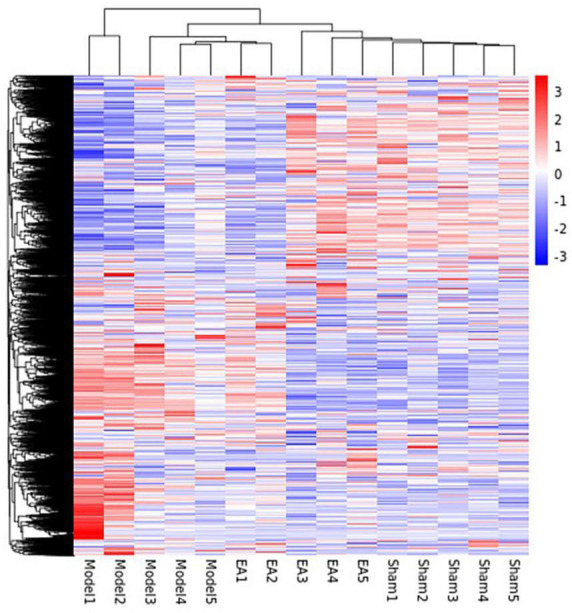
Hierarchical cluster heat map of DEG in each group. This heat map can intuitively compare the homogeneity and differences between groups. Each column in the figure represents a sample, and each row represents a gene. The color in the figure represents the expression amount of the gene in the sample, red represents the high expression amount, and blue represents the low expression amount. The number label next to the color bar at the top left is the specific change trend of the expression amount. On the left is the dendrogram of gene clustering and the module diagram of sub clustering, the closer the two gene branches are, the closer their expression is. The upper part is the dendrogram of sample clustering, and the lower part is the name of the sample. The closer the two sample branches are, the closer the expression pattern of all genes in the two samples is.

**FIGURE 5:**
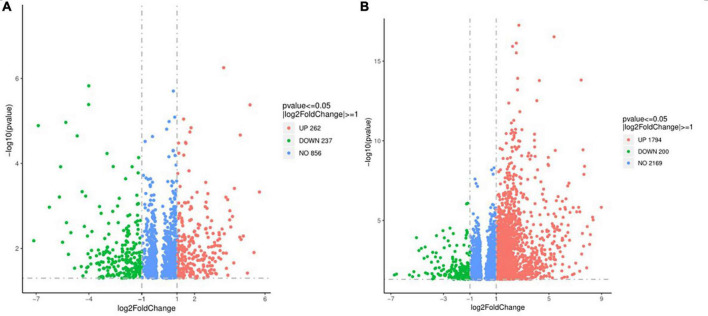
Volcano plot of DEG in each group. **(A)** Volcano plot of DEG between model group and sham group. **(B)** Volcano plot of DEG between model group and EA group. In the figure, the *X*-axis is log2FoldChange, and the *Y*-axis is -log10 (*p*-value). The gray dotted line is the threshold line representing the screening criteria of DEG. Red represents the up-regulated DEG, green represents the down-regulated DEG.

**FIGURE 6:**
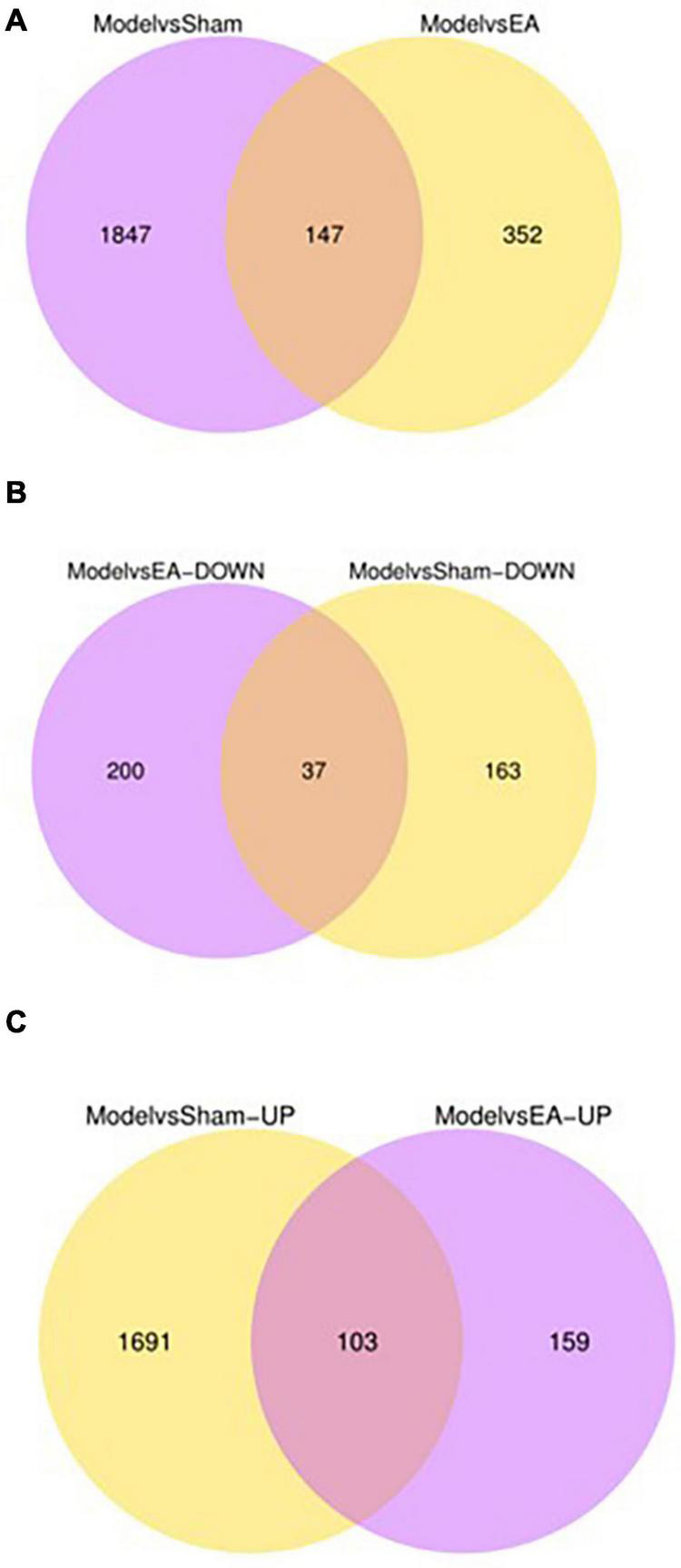
Number of DEG and intersecting genes in each group. **(A)** The purple circle represents the amount of DEG between the model group and the sham group. The yellow circle represents the amount of DEG between the model group and the EA group. **(B)** The purple circle represents the amount of down-regulated DEG between the model group and the EA group. The yellow circle represents the amount of down-regulated DEG between the model group and the sham group. **(C)** The purple circle represents the amount of up-regulated DEG between the model group and the EA group. The yellow circle represents the amount of up-regulated DEG between the model group and the sham group. Overlapping regions represent the intersecting gene between two comparable groups.

The differential expression gene (DEG) in the prostate tissue was analyzed after four courses of EA intervention. The two-dimensional hierarchical cluster heat map showed that samples expression in each group was relatively consistent. Compared with the EA group and sham group, the color of the model group was significantly different, suggesting that the degree of gene expression separation in the model group was significantly different from that in EA group and sham group. The color of the EA group was close to that of sham group, suggesting that the gene expression pattern of EA group was close to that of sham group (Figure 4). The volcano plot showed that there were 1994 intersecting DEGs between model group and sham group (*p* < 0.05, Pmodel/sham), of which 1,794 were upregulated and 200 were downregulated; these DEGs may be related to the modeling of rats. There were 499 intersecting DEGs between EA group and model group (*p* < 0.05, PEA/model), of which 262 were upregulated and 237 were downregulated (Figure 5). The intersection DEG was further analyzed. The result showed that there were 147 intersecting DEGs between Pmodel/sham and Pmodel/EA (*p* < 0.05, Pmodel/sham∩PEA/model), of which 103 intersecting DEG between the upregulated genes in Pmodel/sham and the upregulated genes in Pmodel/EA and 37 intersecting DEG between the downregulated genes in Pmodel/sham and the downregulated genes in Pmodel/EA suggested that EA could reverse the up-/downregulation of these genes in CP/CPPS model rats (Figure 6). These results suggested that the analgesic effect of EA on CP/CPPS rats may be associated with gene expression in prostate tissue.

#### Analysis results of Gene Ontology and Kyoto Encyclopedia of Genes And Genomes

##### Analysis results of Gene Ontology function

**FIGURE 7:**
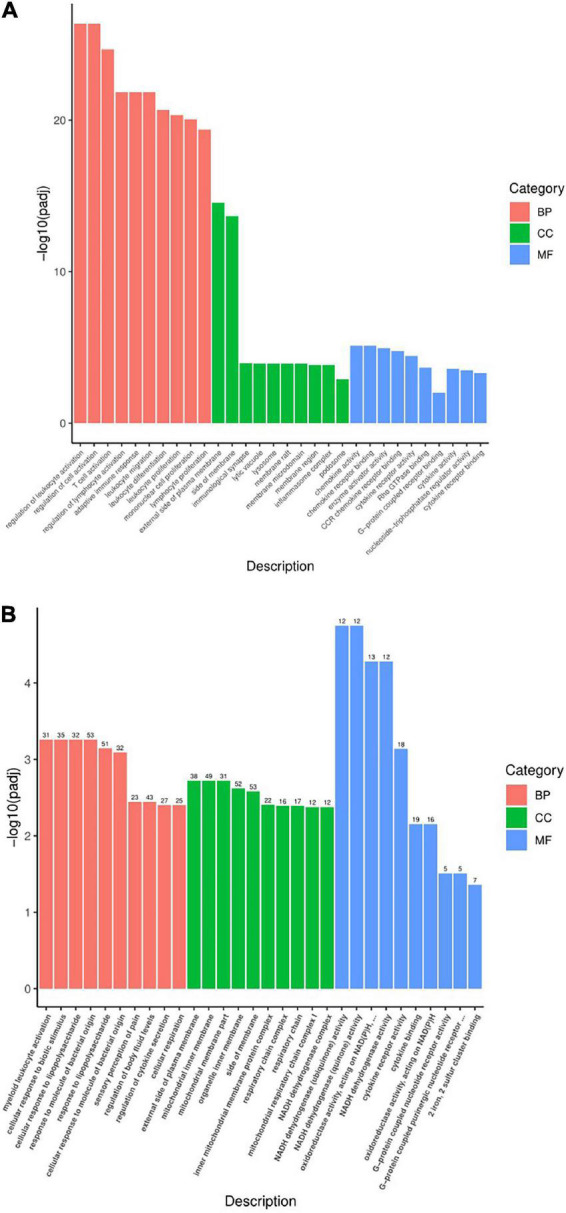
Statistical chart of GO function analysis. **(A)** Statistical chart of GO function analysis of Pmodel/sham. **(B)** Statistical chart of GO function of Pmodel/EA. The *X*-axis represents the secondary classification term of GO, and the *Y*-axis represents the percentage of genes included in the secondary classification. Three colors represent three categories: Red refers to BP (biological process), green refers to CC (cell composition), and blue refers to MF (molecular function).

Gene ontology (GO) function mainly includes three parts: biological process (BP), cell composition (CC), and molecular function (MF). To illustrate the DEG function of rats after modeling, the GO function of Pmodel/sham was analyzed. The results showed that the BP mainly included leukocyte activation regulation, T cell activation regulation, lymphocyte activation regulation, adaptive immune response, leukocyte differentiation, monocyte proliferation, inflammatory response, etc. The CC mainly included the outer side of the plasma membrane, the side of the mold, synapse, plasma membrane receptor complex, the inflammatory body complex, lysosome, etc. The MF mainly included chemokine activity, chemokine receptor binding, cytokine activity, cytokine receptor binding, G protein-coupled receptor binding, actin binding, etc. (Figure 7A).

To illustrate the DEG function of CP/CPPS rats after EA intervention, the GO function of PEA/model was carried out. The results showed that BP mainly included myeloid leukocyte activation, cell response to biological stimulation, cell response to lipopolysaccharide, response to bacterial-derived molecules, pain response, regulation of body fluid level, regulation of cytokine secretion, cell respiration, etc. The CC mainly included outer plasma membrane, motor cartilage membrane, organelle membrane, mitochondrial protein inner membrane complex, mitochondrial respiratory chain complex, respiratory enzyme complex, respiratory chain complex, etc. The MF mainly included cytokine receptor activity, cytokine binding, chemokine activity, oxidoreductase activity, G protein-coupled receptor binding, dehydrogenase activity, etc. (Figure 7B).

What was worth noting was that Pmodel/sham and PEA/model have similar intersecting GO functions, of which the BP mainly included immune response and inflammatory response; the CC mainly included plasma membrane, organelle membrane and other membrane surfaces; the MF is mainly involved in the functions of inflammation and immunity, such as cytokine activity and chemokine activity. The results suggested that the analgesic mechanism of EA on CP/CPPS may be related to the process and function of immune inflammation and the composition of the membrane (Table 2).

**TABLE 2:** Similar GO function analysis between Pmodel/sham and PEA/model.

GO function	Pmodel/sham ∩ PEA/model
BP	Immune cell activation, other immune and inflammatory reactions
CC	Cell membrane, cell organelles membrane
MF	Cytokine activity, chemokine activity, other functions related to immunity and inflammation

Pmodel/sham, GO function analysis results of DEG between model group and sham group. PEA/model, GO function analysis results of DEG between EA group and model group. Pmodel/sham∩PEA/model, similar GO function analysis results between Pmodel/sham and PEA/model.

##### Analysis results of Kyoto Encyclopedia of Genes and Genomes enrichment

**FIGURE 8:**
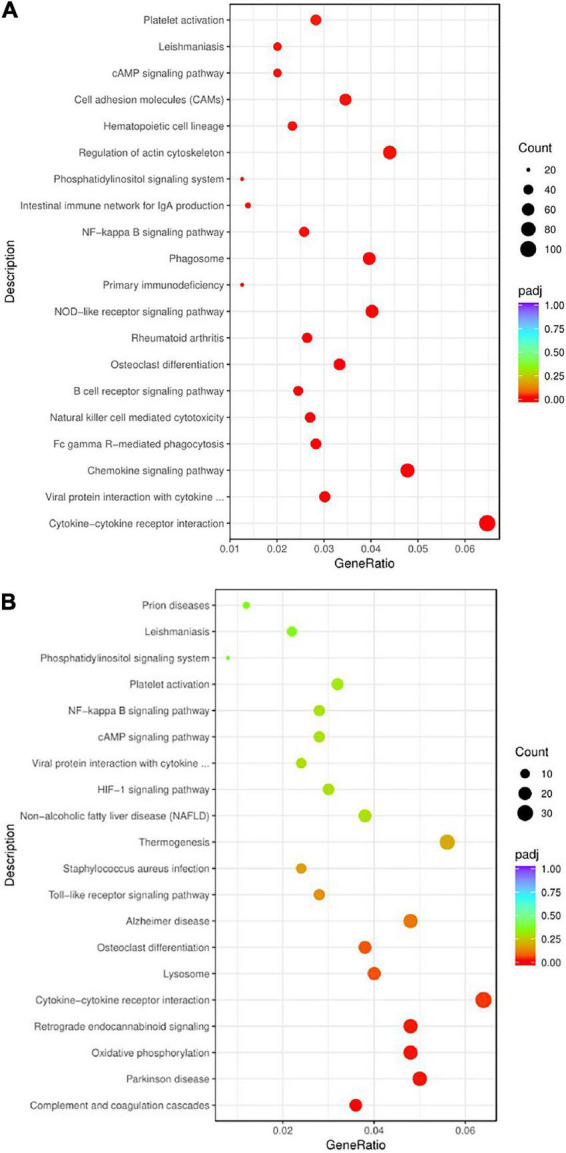
The bubble chart of KEGG enrichment analysis. **(A)** KEGG enrichment analysis bubble chart of Pmodel/sham. **(B)** KEGG enrichment analysis bubble chart of Pmodel/EA. The *Y*-axis represents the pathway name, and the *X*-axis represents the enrichment factor, that is the ratio of the amount of DEG enriched in the KEGG term to the total amount of differential genes. The greater the ratio, the greater the degree of enrichment. The size of the bubble indicates the number of genes in this pathway, and the color of the bubble from red to purple represents the significance of enrichment.

To illustrate the signaling pathways of rats after modeling, Kyoto Encyclopedia of Genes and Genomes (KEGG) enrichment analysis was performed on the Pmodel/sham. The results showed that the pathways mainly included chemokine signal pathway, B-cell receptor signal pathway, NOD-like receptor signal pathway, NF-κB signal pathway, phosphatidylinositol signal pathway, cAMP signal pathway, cytokine–cytokine receptor interaction, rheumatoid arthritis, primary immunodeficiency disease, platelet activation, etc. (Figure 8A).

To illustrate the pathways of CP/CPPS rats after EA intervention, KEGG enrichment analysis was carried out on PEA/model. The results showed that the pathways mainly included Toll receptor signal pathway, NF-κB signal pathway, cAMP signal pathway, phosphatidylinositol signal pathway, complement coagulation cascade, cytokine receptor interaction, staphylococcus aureus infection, non-alcoholic fatty liver disease, platelet activation, Parkinson’s disease, Alzheimer’s disease, osteoclast differentiation, etc. (Figure 8B).

Both of Pmodel/sham and PEA/model were involved in NF-κB signaling pathway, cAMP signaling pathway, phosphatidylinositol signaling pathway, B-cell receptor signaling pathway, chemokine signaling pathway, cytokine receptor interaction, and other immune-related pathways, as well as some immune and inflammatory-related diseases, such as rheumatoid arthritis, primary immune deficiency diseases, staphylococcus aureus infection, and non-alcoholic fatty liver disease (Table 3). The results suggested that the analgesic mechanism of EA on CP/CPPS may be related to the key genes in these intersecting signaling pathways.

**TABLE 3:** Similar KEGG enrichment results between Pmodel/sham and PEA/model.

KEGG enrichment analysis	Pmodel/sham ∩ PEA/model
Signal pathway	NF-kB signaling pathway, cAMP signaling pathway, phosphatidylinositol signaling pathway, B-cell receptor signaling pathway, chemokine signaling pathway, cytokine receptor interaction and other immune-related pathways
Disease	Rheumatoid arthritis, primary immunodeficiency disease, Staphylococcus aureus infection, non-alcoholic fatty liver and other diseases related to immunity and inflammation

Pmodel/sham, KEGG enrichment analysis results of DEG between model group and sham group. PEA/model, KEGG enrichment analysis results of DEG between EA group and model group. Pmodel/sham∩PEA/model: similar KEGG enrichment analysis results between Pmodel/sham and PEA/model.

### Results of qRT-PCR

**FIGURE 9:**
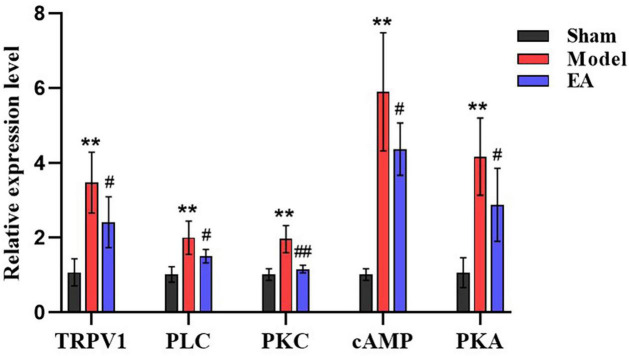
Results of qRT-PCR in each group (*n* = 6, $\bar{x}$ ± s). The *X*-axis is the gene name and the *Y*-axis is the relative expression of the gene. Compared with the sham group, **P < 0.01. Compared with the model group, ^#^*P* < 0.05 and ^##^*P* < 0.01.

**FIGURE 10:**
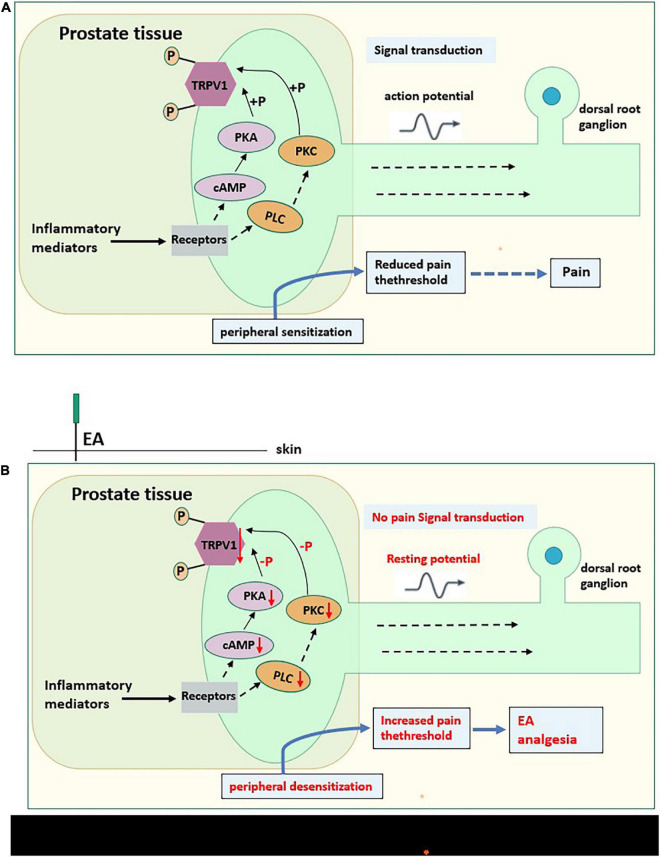
**(A)** The possible Pain-related mechanism diagram of CP/CPPS. Prostate tissue of CP/CPPS model rat produces a variety of inflammatory mediators (sensitizers), which act on homologous receptors expressed by nociceptors to activate the cAMP-PKA pathway and PLC-PKC pathway of intracellular signal transduction. These pathways can phosphorylate TRPV1, causing peripheral sensitization, converting chemical signals into electrical signals, then transmitting to the central system, and reducing the pain threshold. **(B)** The possible analgesic mechanism diagram of EA on CP/CPPS rats. EA on acupoints may interfere with the activation of the cAMP-PKA pathway and PLC-PKC pathway, hindering intracellular signal transduction, reducing the phosphorylation of TRPV1, then causing peripheral desensitization, increasing the pain threshold, and do not transmit noxious stimulation to the central system, to effectively for pain relief. “_⇢_”Directly acting on downstream substances,“_→_” Indirect action on downstream substances.

To verify the RNA-Seq results and further scientifically study the related signal pathways involved in the analgesic effect of EA on CP/CPPS. Based on the results of RNA-Seq analysis in prostate tissue of rats in each group, and combined with the modern research progress on the pain-related mechanism of CP/CPPS, our team screened out the pain-related DEG and the key genes in pain-related pathways, vanillic acid receptor subtype 1 of the transient receptor potential (TRPV1), phospholipase C (PLC), protein kinase C (PKC), cyclic adenosine monophosphate (cAMP), and protein kinase A (PKA). qRT-PCR results showed that compared with sham group, TRPV1, PLC, PKC, cAMP, and PKA in model group were increased significantly (*p* < 0.01). Compared with the model group, TRPV1, PLC, PKC, cAMP, and PKA in EA group were decreased (*p* < 0.05, *p* < 0.01) (Figure 9). qRT-PCR results suggested that the analgesic effect of EA on CP/CPPS may be achieved by regulating the expression of TRPVI, PLC, PKC, cAMP, and PKA (Figures 10A,B).

## Discussion

Pain is a major symptom in patients with CP/CPPS (Lin et al., 2016). Most clinical studies have shown that EA is an effective means of improving pain symptoms in CP/CPPS patients, because it is inexpensive, safe, has few side effects and has a long-lasting efficacy (Sun et al., 2021). Combined with medication, it can significantly improve the clinical efficacy of the medication, including improving various symptoms and indicators in CP/CPPS patients (Liang et al., 2021). Our previous studies (Wu et al., 2022; Xu et al., 2022) also assessed the effects of EA on the behavioral pain profile of CP/CPPS rats. The result showed that EA was effective in reducing mechanical and thermal pain in CP/CPPS rats; it also revealed a decrease in serum substance P (SP) and prostaglandin E2 (PGE2) and a decrease in cyclooxygenase-2 (COX-2) and PGE2 in prostate tissue, suggesting that the analgesic mechanism of EA in CP/CPPS rats may be related to the modulation of the body’s inflammatory response. This study has investigated the analgesic mechanism of EA therapy in CP/CPPS rats from the prostate transcriptome level. As a peripheral injury receptor in CP/CPPS rats, the prostate tissue is a key site for sensing and transmitting nociceptive stimuli. Various causative factors can cause sensitization of pain throughout the pelvis by stimulating sensory nerves in the prostate tissue and generating pain signals (Jiang et al., 2021). In the H&E staining results of this study, we found that EA significantly improved the histopathological changes of prostate tissue in CP/CPPS rats, suggesting that the effect of EA on CP/CPPS may be related to the protective effect on prostate tissue. This is one of the reasons why we investigated the analgesic mechanism of EA on CP/CPPS from the transcriptome level of the prostate tissue.

**FIGURE 11:**
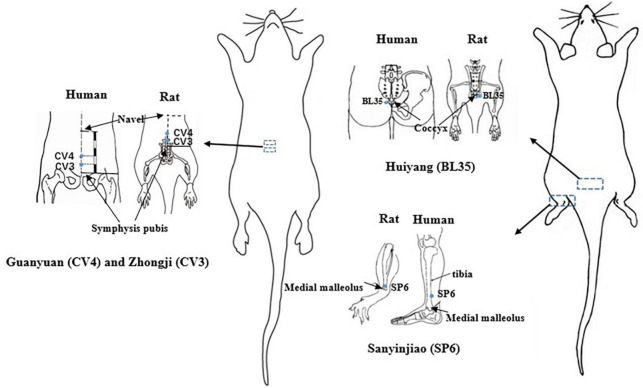
Schematic representation of the points in the EA group. Anatomical localization of the acupoints are shown on the rat and human bodies. Points are indicated by blue dots.

The acupoints used in this study included CV4, CV3, SP6 and BL35 (Figure 11). A meta-analysis showed that these acupoints are the most frequently used acupoints in the clinical treatment of patients with CP/CPPS (Zhang et al., 2021). In terms of Traditional Chinese Medicine (TCM) theory, CV3 and CV4 are acupoints on the Ren Vessel, which have a function of warming Yang and dredging collaterals (Zheng and Hu, 2017; Ying H. Z. et al., 2021). SP6 is the intersection acupoint on Three Yin Meridian of Foot, which has the functions of soothing liver and regulating Qi, fortifying the spleen and disinhibiting dampness, warming kidney and tonifying Qi (Ying Z. H. et al., 2021). BL35 is on the Bladder Meridian of Foot Taiyang, which has the function of promoting Qi and water (Liu et al., 2016). The combination of the above acupoints can relieve CP/CPPS pain by warming Yang, promoting Qi and disinhibiting dampness. In addition, from modern neuroanatomy, the prostate is innervated by the parasympathetic nerves, and the painful areas of CP/CPPS patients are mainly distributed by the pubic nerve, perineal nerve and pelvic nerve, and these nerve fibers mainly originate from the lumbar and sacral plexuses of the spinal cord segments (Wang and Shang, 2010). The sympathetic and parasympathetic nerves are distributed under the CV3 and CV4, and some studies (Chang et al., 2009) have shown that by stimulating CV3 and CV4, it can relieve pelvic and prostate pain. This can relieve the muscle tension around the pelvis and prostate, promote local blood circulation and lower PGE2 levels, thereby relieving the stimulation of parasympathetic and sympathetic afferent fibers by these substances and reducing the transmission of undesirable impulses to the center, thus reducing the painful symptoms of chronic prostatitis. The tibial nerve is located under the SP6, and some studies have shown (Abdel-Karim et al., 2005) that electrical stimulation of SP6 allows impulses to reach the posterior roots of the spinal cord through the tibial nerve, which regulates the function of the corresponding organs through central feedback. Stimulation of SP6 may regulate prostate activity through tibial nerve conduction. BL35 is in the projection area of the prostate, which has caudal nerves in the superficial part and pudendal nerve trunks in the deep part; acupuncture at BL35 can regulate the excitability of the pudendal and pelvic nerve (Li et al., 2009). From the above, it can be seen that the nerves under CV4, CV3, SP6, and BL35 overlap with those of the CP/CPPS pain site. The analgesic effect of EA on CP/CPPS at CV3, CV4, SP6, and BL35 may be related to the corresponding relationship between the nerves distributed at these acupoints and the nerves distributed at the pain site of CP/CPPS.

RNA-Seq allows the study of gene function and gene structure at a holistic level, revealing specific biological processes and molecular mechanisms in the development of disease (Lv et al., 2020). In this study, there were 147 intersecting DEG between Pmodel/sham and Pmodel/EA, of which 103 were upregulated and 37 were downregulated (Supplementary Table 1), indicating that EA can reverse the up-/downregulated genes in prostate tissue of CP/CPPS rats. These intersecting DEGs may be closely related to the analgesic mechanism of EA on CP/CPPS rats.

Comprehensively comparing the GO analysis of Pmodel/sham and PEA/model, there is a crossover of GO functions between Pmodel/sham and PEA/model (Table 2). The results of GO analysis suggest that the analgesic effect of EA on CP/CPPS may be related to inflammation-related ion channels in the cell membrane of prostate tissue. In the process of inflammatory pain, the inflammatory reaction will make the nociceptor sensitive, resulting in the decrease of activation threshold, and the pain hypersensitivity at the inflammatory site (Liu and Zhang, 2009). The largest receptor that acts as noxious stimulus detectors in injury receptors are the transient receptor potential ion channel protein (TRP) family. TRP channels are expressed on cell membranes and mediate non-selective cation inward flow by integrating intra- and extracellular information (Szallasi et al., 2007; Darré and Domene, 2015). TRPV1 is a subtype of TRP channels whose physiological function primarily responds to inflammatory pain stimuli and is involved in nociceptive signaling (Julius, 2013). The binding of inflammatory mediators to TRPV1 in peripheral tissues enhances cell membrane excitability, decreases inflammatory pain thresholds and causes nociceptive sensitization (Zhang and Wang, 2017). In modern medical research, several studies (Caterina et al., 2000; Davis et al., 2000) found that TRPV1 gene-deficient mice can reduce inflammatory mediator-induced nociceptive sensitization. Several studies (Lu et al., 2016) have also shown that effective relief of inflammatory pain by EA is associated with the modulation of TRPV1. In the present RNA-Seq results of CP/CPPS rat prostate tissue, we also found TRPV1 to be an upregulated gene in Pmodel/sham and a downregulated gene in PEA/model (Supplementary Table 1). qRT-PCR results also found that TRPV1 was significantly upregulated in the model group and significantly downregulated after EA intervention, in line with the trend of RNA-Seq results, suggesting that the analgesic effect of EA on CP/CPPS may be related to the regulation of TPPV1.

In the results of KEGG enrichment analysis, Pmodel/sham intersected with the pathways mainly involved in PEA/model (Table 3), and the results suggest that the effect of electrodes on CP/CPPS rats may be related to the regulation of phosphatidylinositol signaling pathway and cAMP signaling pathway. In the phosphatidylinositol signal pathway, extracellular signaling molecules can bind to G protein-coupled receptors to activate PLC, which then indirectly activate PKC, and regulate downstream signaling molecules (Yi et al., 2021). Recent studies (Bai, 2018; Xue et al., 2021) have shown that PLC-PKC pathway plays a significant role in chronic pain generation and maintenance. In the cAMP signaling pathway, recent studies (Smith et al., 2000; Joseph and Levine, 2003) have shown that pain hypersensitivity in rats, an inflammation model, is closely associated with the cAMP-PKA pathway. In this qRT-PCR results, PLC, PKC, cAMP, and PKC increased significantly in the model group and significantly decreased after EA intervention. The results were consistent with the KEGG enrichment analysis, suggesting that the analgesic effect of EA on CP/CPPS may be related to the modulation of the cAMP-PKA and PLC-PKC signaling pathways.

Recent studies (Bhave et al., 2002; Mohapatra and Nau, 2005) also have suggested that the TRPV1 phosphorylation mediated by cAMP-PKA and PLC-PKC signaling pathways may be the mechanism of inflammatory pain. In the present qRT-PCR results, PLC, PKC, cAMP, PKA, and TRPV1 in the model group were significantly increased and significantly decreased after EA intervention, suggesting that the analgesic effect of EA on CP/CPPS may be achieved by reducing the TRPV1 phosphorylation mediated by cAMP-PKA and PLA-PKC signaling pathways.

In conclusion, the present study is the first to apply RNA-Seq technology to the study of the analgesic effect of EA on CP/CPPS. The results indicate that the analgesic effect of EA on CP/CPPS involves a variety of GO biological functions and multiple signaling pathways. However, some questions still need to be addressed in this study. The study did not investigate how EA affects cAMP, PKA, PLC, PKC, TRPV1 and other important genes in the cAMP signaling pathway and PKC signaling pathway, and the specific targets of EA intervention on CP/CPPS need to be further investigated. In the KEGG enrichment analysis results, the DEGs were also enriched in the NF-κB pathway and other immune-related pathways in the model rats after EA intervention. Whether the analgesic effects of EA at CV3, CV4, SP6, and BL35 on CP/CPPS are related to these pathways and their extensiveness also need to be further investigated in the future.

## Materials and methods

### Animals

Thirty male Sprague–Dawley (SD) rats of 7–8 weeks’ weight (210 ± 10 g) were fed in the laboratory of Beijing University of Chinese Medicine (SPF level), with food and water freely. All rats were provided by *Beijing Vitong Lihua Laboratory Animal Technology Co., Ltd*. The feeding temperature was 23 ± 2°C, the humidity was 45%, and the light and dark periods were 12 h (turning on the light at 8 a.m.). Adaptive feeding occurred for 1 week. Rats were randomly divided into sham group (*n* = 10), model group (*n* = 10) and EA group (*n* = 10). Ethical approval was granted by the Beijing University of Chinese Medicine (BUCM-3-20151202-4001); therefore, experiments were performed in accordance with the ethical standards laid out by the IACUC.

### Modeling methods

Referring to the modeling method of Tang (2007), fasting and water deprivation occurred 24 h before modeling. Rats were anesthetized by intraperitoneal injection of 1% pentobarbital sodium (350 mg/kg body weight). All rats were in the supine position, cut off the hair on the right lower abdomen, and a longitudinal incision (1 cm beside the anterior midline) was made to expose the bilateral ventral lobes of the prostate. Stabilizing the prostate with hemostatic forceps, the rats in model group and EA group were injected with 0.05 mL of complete Freund’s adjuvant (CFA) solution (Beijing Benovir Biotechnology Co., Ltd.) into the ventral lobes of the prostate on both sides, while the rats in sham group were injected with the same dose of saline. Then layer by layer disinfection, suturing, and disinfection. All rats were kept in a warm place until awake. All of the rats fasted for 24 h postoperatively, but free water was allowed. Pathological changes of prostate tissues were observed by H&E staining to evaluate the successful model. Under the microscope, a large number of inflammatory cell infiltration in the prostate gland was to prove the successful establishment of the model. All experiment procedures were performed according to the National Institutes of Health Guide for the Care and Use of Laboratory Animals.

### Intervention methods

In sham and model groups, the rats were bounded and fixed every day without other treatment. In EA group, firstly, stainless steel acupuncture needles (0.18 × 13 mm, *Beijing Zhongyan Taihe Medical Instrument Co., Ltd.*) were inserted at a depth of 3 mm into the CV4 (located at points 3/5 down the ventral midline connecting the umbilicus to the pubic tubercle (Huang, 2020)), CV3 (located at points 4/5 down the ventral midline connecting the umbilicus to the pubic tubercle (Huang, 2020)) and bilateral SP6 (located at 10 mm proximal to the highest prominence of the medial malleolus, on the posterior border of the medial crest of the tibia (Wu et al., 2019)) in the supine position, 20 min for each time; then, bilateral BL35 (located at anteromedial of the transverse process of the 6th lumbar spine (Liu and Wang, 2013)) was inserted at a depth of 6 mm inward toward the spine in the prone position, 20 min for each time. The two ipsilateral needles were connected to the output terminals of the HANS-200E EA instrument with 2 Hz/100 Hz alternating frequencies. The output current was set as 2–3 A, with the tail of the rats swinging slightly without obvious struggle. All groups were treated on the 15th day after modeling. The treatment was once a day for 40 min, 5 days as a course, with 2 days of rest between the two courses, 4 courses in total. Before the formal intervention, the animals were stroked for 5 min every day, and all rats were loosely fixed throughout the treatment (Figures 1, 11).

### Sample collection

The day after the last treatment, all rats were anesthetized with an intraperitoneal injection of 1% pentobarbital sodium (350 mg/kg body weight). The lower abdominal skin of rats was cut to fully expose the prostate. After carefully stripping and removing the prostate, the left lobe of the prostate was fixed in 4% paraformaldehyde, and the right lobe of the prostate was stored in a refrigerator at 80°C.

### Histological examination

The prostate tissue fixed in 4% paraformaldehyde was dehydrated in a fully automatic dehydrator and then embedded in paraffin and sectioned at a thickness of 4 μm. Paraffin sections were dewaxed with xylene, hydrated with alcohol, stained with hematoxylin and eosin, dehydrated with alcohol and made transparent with xylene. The morphology of the prostate tissue was observed under an optical microscope.

### RNA extraction and construction of sequencing library

Five frozen right lobes of the prostate tissues were taken from each group for processing and sequencing (*n* = 5), Trizol (Invitrogen, United States) method was used to extract total RNA, and the quality inspection of the obtained total RNA was performed using Agilent 2100 bioanalyzer to observe its purity, concentration and integrity. For library establishment, the library was constructed of qualified samples with a starting quantity of 1 μg total RNA ≥ 50 ng/μL. PCR amplification was performed to obtain the final cDNA library. The Agilent 2100 bioanalyzer was used to check the library quality. The transcriptome sequencing was completed based on the HiSeq sequencing platform. The illumine PE library was constructed by Illumina TruSeq TM RNA Sample Prep Kit method for 2 × 150 bp sequencing. The quality control of the sequencing data was carried out, and then the transcriptome data were analyzed by bioinformatics. Fragments per kilobase per million mapped reads (FPKM) value was used to determine gene expression level. DEG was analyzed with the full transcriptomic data of each sample.

### Gene ontology and Kyoto Encyclopedia of Genes and Genomes analyses

GO terms are used to describe and classify the functions of genes and proteins. When the corrected *p*-value < 0.05, it is considered that the GO function is significantly enriched. KEGG is a large knowledge base for systematic analysis of gene function and association of genomic information. Corrected *p*-value < 0.05 was used to identify which pathway is significantly enriched in DEG compared with the whole genome background.

### qRT-PCR

Total RNA was extracted using HiPure Total RNA Mini Kit (Guangzhou Magen Biotechnology Co., Ltd.). RNA was quantified by spectrophotometry. The First-Strand Synthesis Master Mix (Beijing LABLEAD Biotech Co., Ltd.) was used for reverse transcription to obtain cDNA. Premier 5.0 software (Premier, Canada) was used to design specific oligonucleotide primers for rat TRPV1, PLC, PKC, cAMP, PKA and GAPDH (as an internal reference gene). The primers listed were amplified (Table 4). The reaction mixture (total volume 10 μL) was prepared using cDNA (1 μg/μL), forward and reverse primers (0.5 μL), PowerUpTMSYBRTM Green Master Mix (5 μL) (Life Technologies) and Nuclease-Free Water (3 μL). The amplification was carried out with an initial denaturation step at 95°C for 3 min, followed by 44 repeated thermal cycles (95°C for 30 s, 60°C for 30 s, 72°C for 30 s, 95°C for 2 min). The relative quantification of TRPV1, PLC, PKC, cAMP, PKA normalized to GAPDH and relative to a calibrator was measured by 2^–ΔΔ^ *^Ct^*.

**TABLE 4:** qRT-PCR primers.

Gene	Primer name	Sequence (5′–3′)
1	TRPV1-F	AGAAGGGGAACCAGGGCAAAG
	TRPV1-R	TCAACGAGGACCCAGGCAACT
2	PLC-F	AAGCCTTTGACCCCTTTGAT
	PLC-R	CCAGCCACTTCAATCTCCAC
3	PKC-F	TCTGGAAGCAGCAATAGAGTT
	PKC-R	TCATCAAGGTGTTAGGCAAAG
4	cAMP-F	CCCTGAACTCAACTGTGAAATAGCA
	cAMP-R	CCCAAGTCAAGGGCTTGGAA
5	PKA-F	ACCTTGGGAACGGGTTCCTTCG
	PKA-R	TACACCCAATGCCCACCAGTCC
GAPDH	GAPDH-F	ACCACAGTCCATGCCATCAC
	GAPDH-R	TCCACCACCCTGTTGCTGTA

### Statistical analysis

IBM SPSS 20.0 software was used for statistical analysis of the data. The data in the normal distribution were represented as the mean ± standard deviation (−x ± s), and the one-way analysis of variance least significant difference method was used for comparison between groups with a homogeneous variance, while Dunnett’s T3 method was used for those with a heterogeneous variance. The data that did not follow a normal distribution were represented by the median and quartile [median (P25, P75)], and non-parametric tests were used for comparison between the groups. The test level was set as α = 0.05, with *p* < 0.05 considered statistically significant.

## Conclusion

After modeling, the expression of some genes in prostate tissue increased or decreased. EA acting on CP/CPPS rats could reduce the upregulated genes expression and increase the downregulated genes expression, so that the genes expression tends toward normal and therefore could relieve pain symptoms of CP/CPPS. The RNA-Seq data obtained in this study have been proven to be verified with qRT-PCR. The analgesic effect of EA on CP/CPPS may be related to the cAMP-PKA-TRPV1/PLC-PKC-TRPV1 signal pathway. This study, for the first time, applied RNA-Seq to analyze the gene expression profiling in prostate tissue after EA intervention and provides theoretical support and scientific basis for the analgesic effect of EA on CP/CPPS in clinics.

## Data availability statement

The data is publicly available at the following link: https://dataview.ncbi.nlm.nih.gov/?archive=bioproject.

## Ethics statement

The animal study was reviewed and approved by the Beijing University of Chinese Medicine (BUCM-3-20151202-4001).

## Author contributions

NL contributed to the conception and design of the study. X-LW wrote the first draft of the manuscript. X-LW, CX, T-HY, Z-WY, Q-HS, and X-HQ performed the experiments. KC, Y-MC, and YT organized the database. WC and J-NZ performed the statistical analysis. X-YY wrote sections of the manuscript. All authors contributed to manuscript revision and read and approved the submitted version.

## Abbreviations

CP/CPPS: chronic prostatitis/chronic pelvic pain syndrome
EA: electroacupuncture
RNA-Seq: transcriptome sequencing
COX-2: cyclooxygenase-2
PGE2: prostaglandin E2
β-EP: β-endorphin
DEG: differential expression gene
CFA: complete Freund’s adjuvant
CV4: “Guanyuan”
CV3: “Zhongji”
SP6: “Sanyinjiao”
BL35: “Huiyang”
GO: Gene Ontology
KEGG: Kyoto Encyclopedia of Genes and Genomes
BP: biological process
CC: cell composition
MF: molecular function
TRP: transient receptor potential ion channel protein
TRPV1: vanillic acid receptor subtype 1 of the transient receptor potential
PLC: phospholipase C
PKC: protein kinase C
cAMP: cyclic adenosine monophosphate
PKA: protein kinase A
SD: Sprague–Dawley.
