# Supplementary material for: Effect of electroacupuncture on cyclic adenosine monophosphate-protein kinase A-vanillic acid receptor subtype 1 of the transient receptor potential/PLK-protein kinase C-vanillic acid receptor subtype 1 of the transient receptor potential pathway based on RNA-seq analysis in prostate tissue in rats with chronic prostatitis/chronic pelvic pain syndrome
Source: Front Neurosci. 2022 Aug 24;16:938200. doi: 10.3389/fnins.2022.938200 (PMC9449126; doi:10.3389/fnins.2022.938200)
Supplement: Supplementary file 1 [file Image_1.pdf]

Supplementary Figures in manuscripts

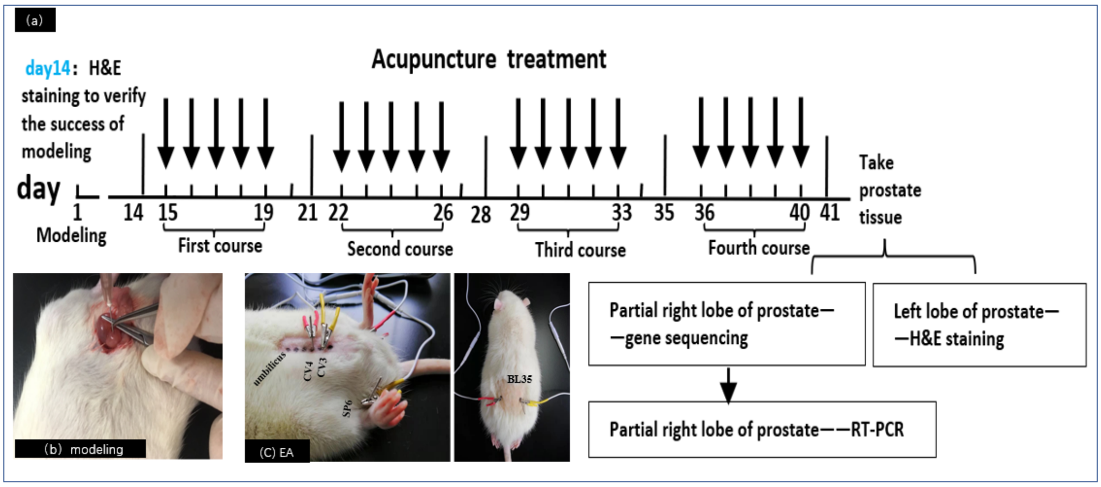

Supplementary Figure 1. The schematic diagram of this experiment. **(a) The** schematic diagram for methodologies. Male SD rats were injected with CFA into the ventral lobes of the prostate on both sides on the first day, and H&E staining was used to judge whether the model was successful or not on the 14th day. All groups were treated on the 15th day after modeling. Treatment once a day for 40 minutes, 5 days as a course of treatment, with 2 days of rest between the two courses, a total of four courses. At the end of the last course of treatment, the rats were anesthetized and sacrificed on the next day and take the prostates. The left lobe of the prostate was stained with H&E, and partial right lobes were analyzed with RNA-Seq technology. Then, combined with the results of RNA-Seq and modern research progress on the pain-related mechanism of CP/CPPS, the DEG and pain-related signal pathways were screened out. Finally, the key genes in the pathways were verified by QRT-PCR. **(b)** Injecting CFA into the prostate of rats to make CP/CPPS model. **(c)** Acupoint location and EA process in CP/CPPS rats. CV3(zhongji), CV4(guanyuan), SP6(sanyinjiao), BL35(huiyang).

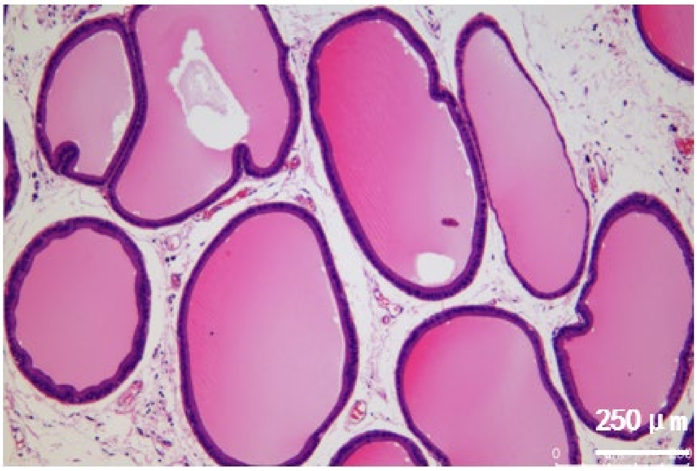

(a)

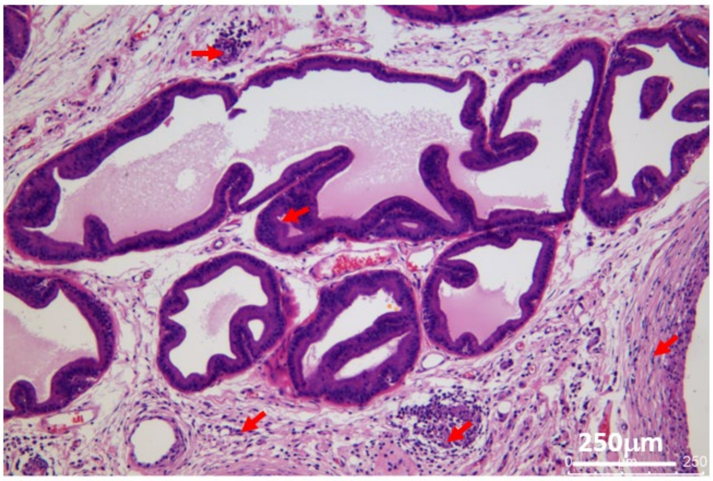

(b)

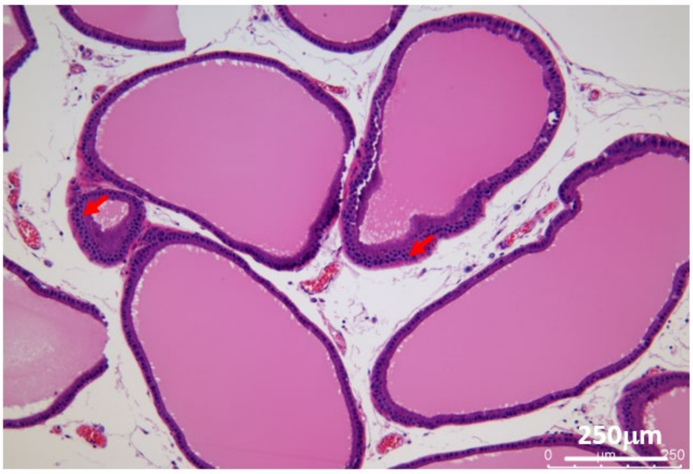

(C)

Supplementary Figure 2. Histological morphology of prostatic tissue in each group. **(a)** The result of H&E staining in sham group; **(b)** The result of H&E staining in model group; **(c)** The result of H&E staining in EA group. Red arrows show the infiltration of inflammatory cells.

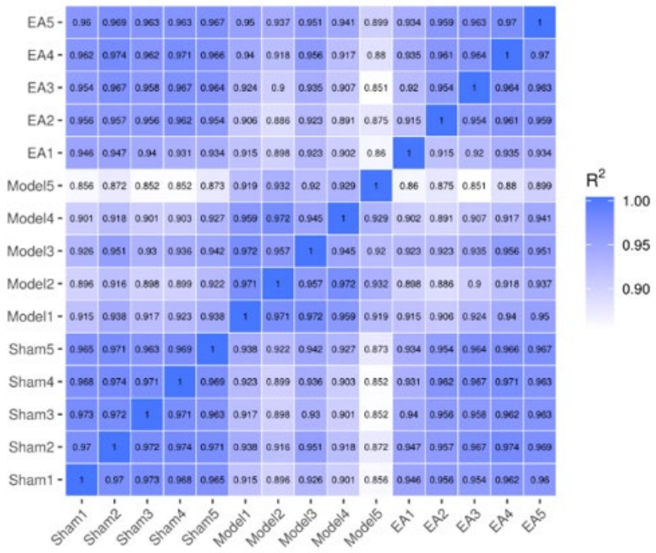

Figure 3. Correlation heat map between samples. The correlation heat map between samples is often used to evaluate the sample difference between groups and the sample repetition within groups. The X-axis and Y-axis in the above figure are sham group (sham1, 2, 3, 4, 5), model group (model1, 2, 3, 4, 5) and EA group (EA1, 2, 3, 4, 5). The values between the X-axis and Y-axis are the square of the correlation coefficient between the corresponding samples. The darker the color indicates the higher the correlation coefficient between samples, and the lighter the color indicates the lower the correlation coefficient between samples.

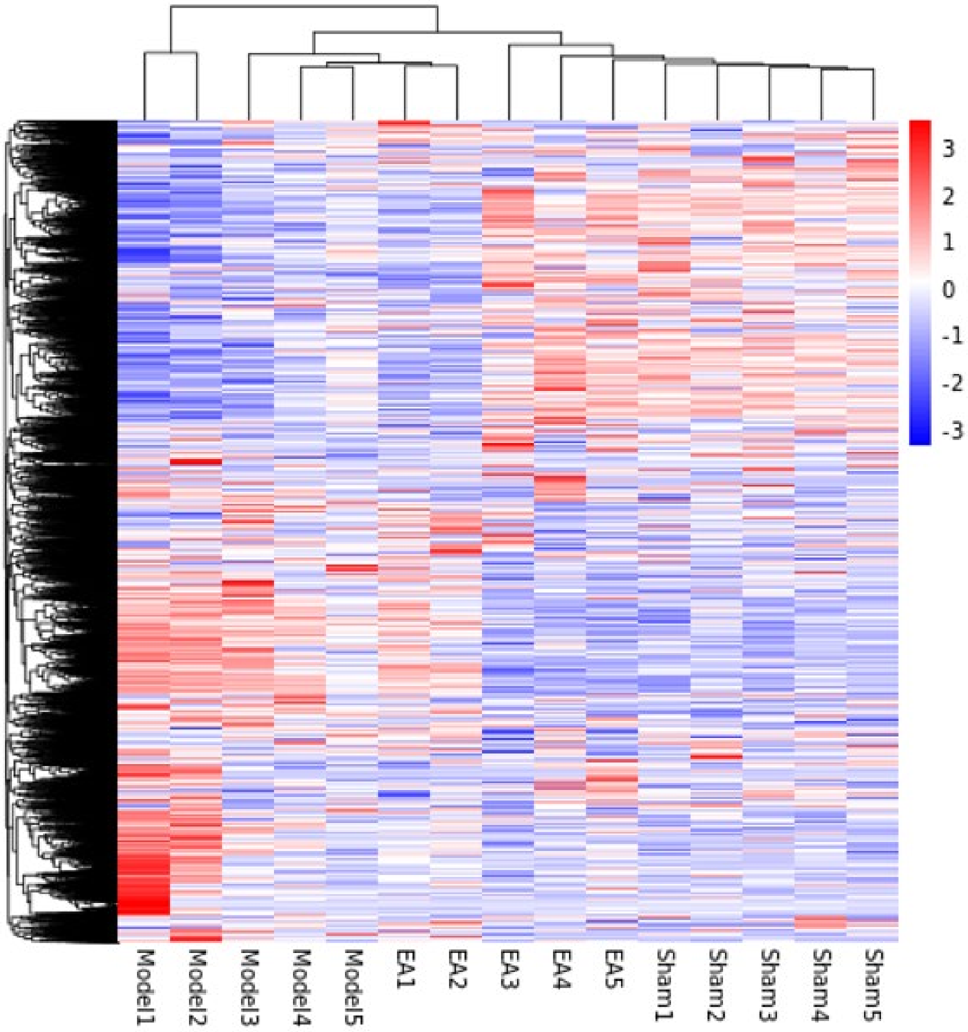

Supplementary Figure 4. Hierarchical cluster heat map of DEG in each group. This heat map can intuitively compare the homogeneity and differences between groups. Each column in the figure represents a sample, and each row represents a gene. The color in the figure represents the expression amount of the gene in the sample, red represents the high expression amount, and blue represents the low expression amount. The number label next to the color bar at the top left is the specific change trend of the expression amount. On the left is the dendrogram of gene clustering and the module diagram of sub clustering, the closer the two gene branches are, the closer their expression is. The upper part is the dendrogram of sample clustering, and the lower part is the name of the sample. The closer the two sample branches are, the closer the expression pattern of all genes in the two samples is.

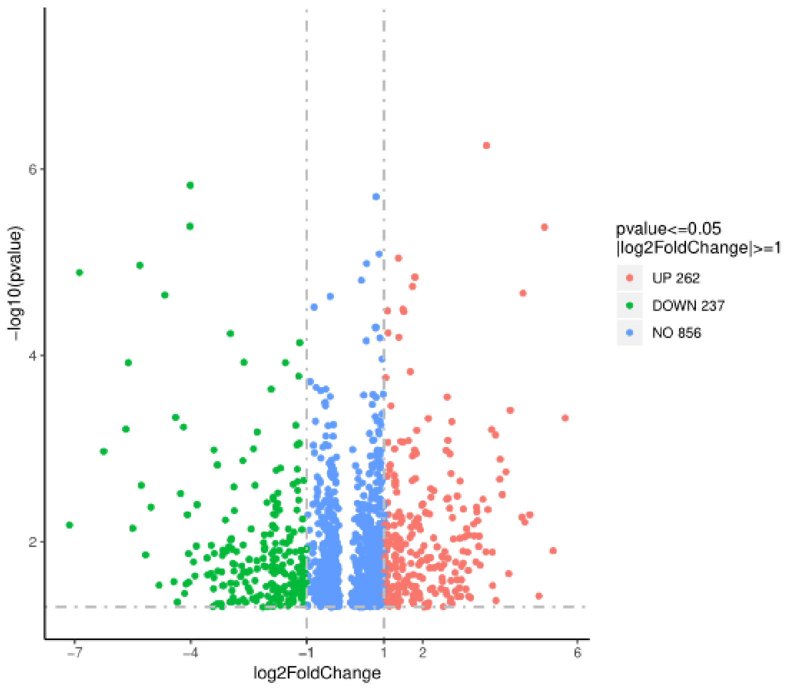

（a）

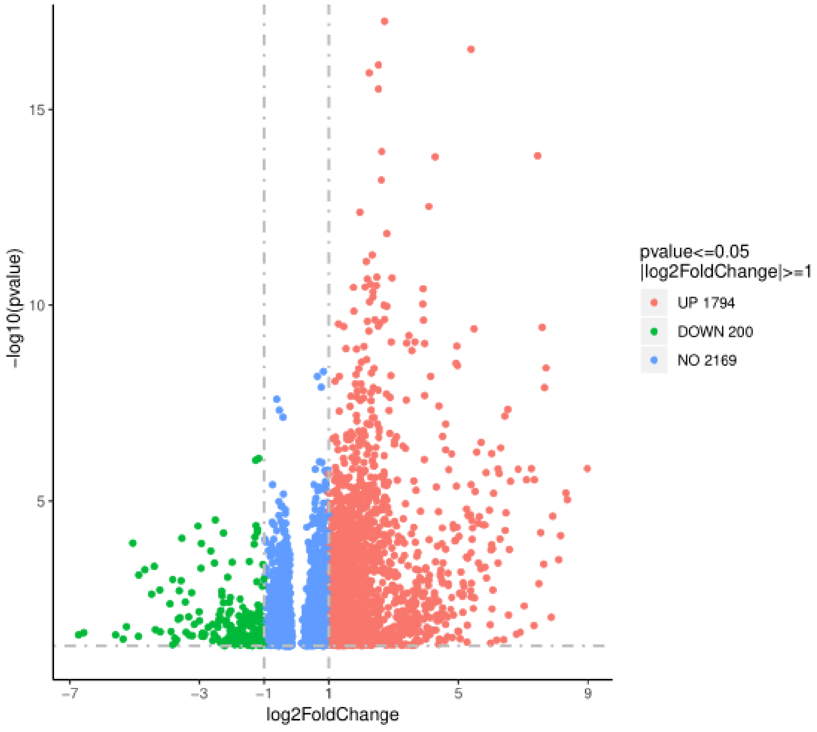

（b）

Supplementary Figure 5. Volcano plot of DEG in each group. **(a)** Volcano plot of DEG between model group and sham group. **(b)** Volcano plot of DEG between EA group and model group. In the figure, the X-axis is log2FoldChange, and the Y-axis is -log10 (pvalue). The gray dotted line is the threshold line representing the screening criteria of DEG. Red represents the up-regulated DEG, green represents the down-regulated DEG.

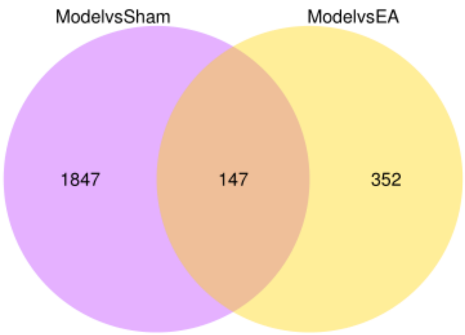

（a）

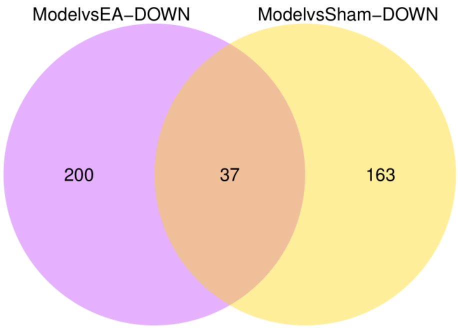

（b）

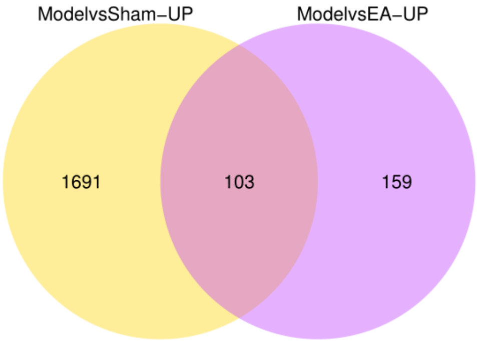

（c）

Supplementary Figure 6. Number of DEG and intersecting genes in each group. **(a)** The purple circle represents the amount of DEG between the model group and the sham group. The yellow circle represents the amount of DEG between the model group and the EA group. **(b)** The purple circle represents the amount of down-regulated DEG between the model group and the EA group. The yellow circle represents the amount of down-regulated DEG between the model group and the sham group. **(c)** The purple circle represents the amount of up-regulated DEG between the model group and the EA group. The yellow circle represents the amount of up-regulated DEG between the model group and the sham group. Overlapping regions represent the intersecting gene between two comparable groups.

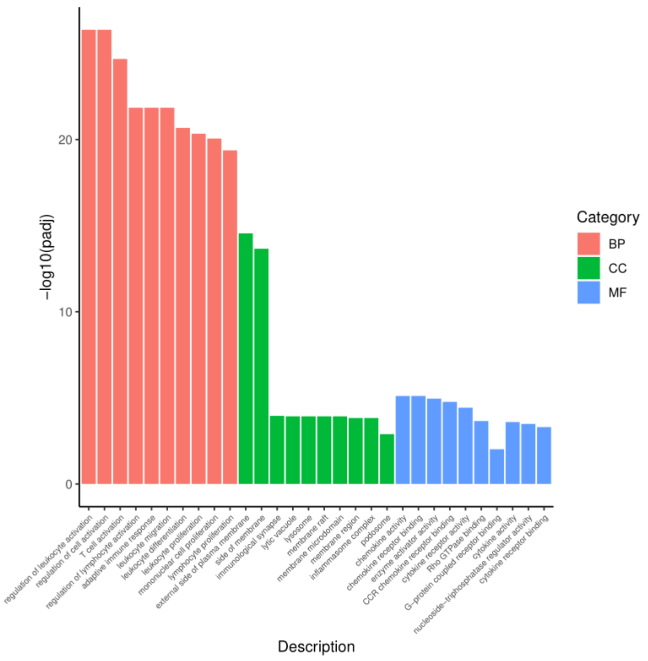

(a)

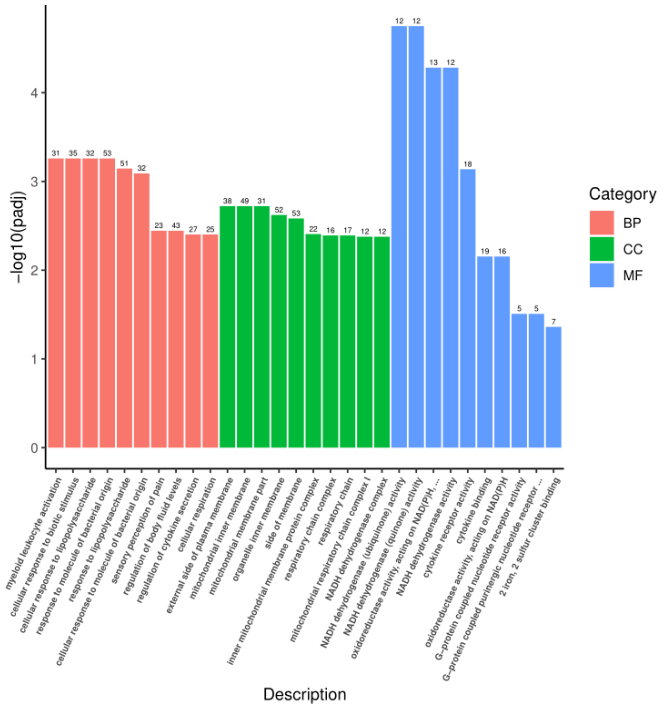

(b)

Supplementary Figure 7. Statistical chart of GO function analysis. **(a)** Statistical chart of GO function analysis of Pmodel/sham. **(b)** Statistical chart of GO function of PEA/model. The X-axis represents the secondary classification term of GO, and the Y-axis represents the percentage of genes included in the secondary classification. Three colors represent three categories: Red refers to BP (biological process), green refers to CC (cell composition), and blue refers to MF (molecular function).

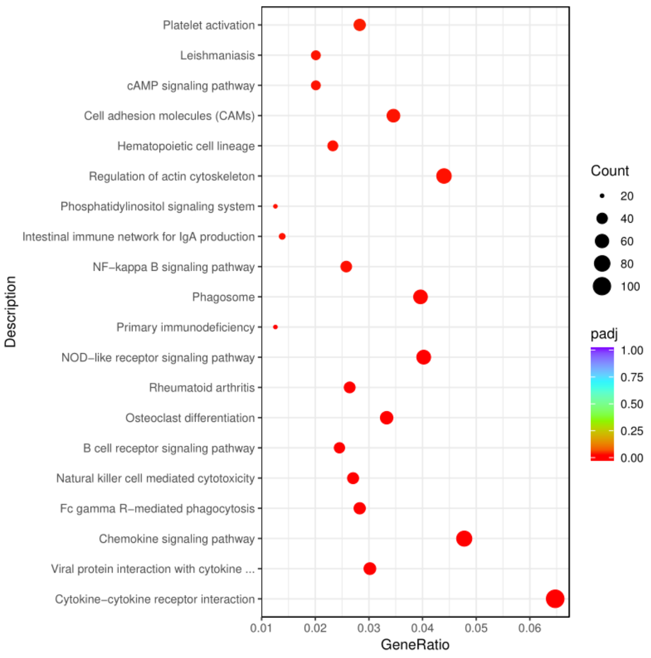

(a)

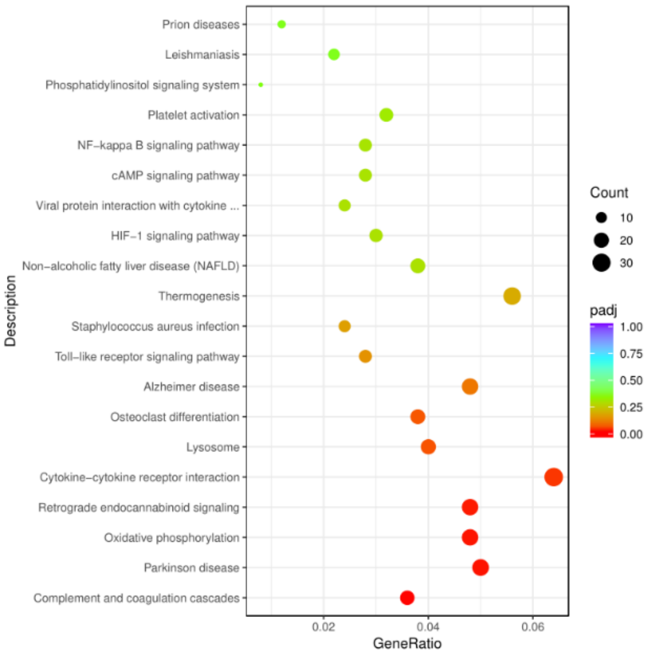

(b)

Supplementary Figure 8. The bubble chart of KEGG enrichment analysis. **(a)** KEGG enrichment analysis bubble chart of Pmodel/sham. **(b)** KEGG enrichment analysis bubble chart of PEA/model. The Y-axis represents the pathway name, and the X-axis represents the enrichment factor, that is the ratio of the amount of DEG enriched in the KEGG term to the total amount of differential genes. The greater the ratio, the greater the degree of enrichment. The size of the bubble indicates the number of genes in this pathway, and the color of the bubble from red to purple represents the significance of enrichment.

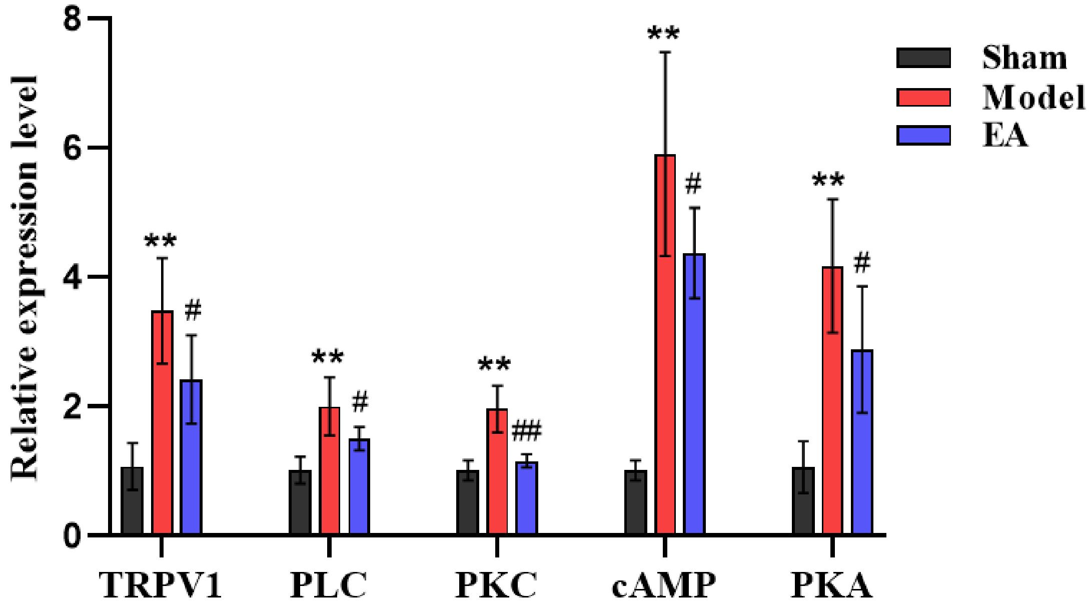

Supplementary Figure 9. Results of QRT-PCR in each group (n=6, $\bar{x}\pm s$). The X-axis is the gene name and the Y-axis is the relative expression of the gene. Compared with the sham group, $^{**}P<0.01$. Compared with the model group, $^{\#}P<0.05$, $^{\#\#}P<0.01$.

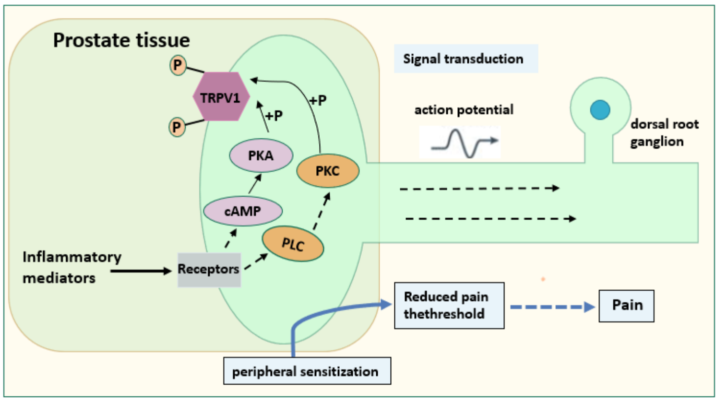

（a）

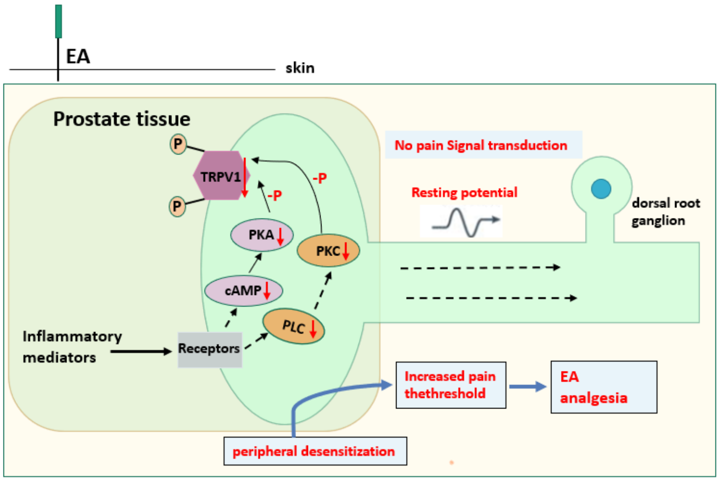

（b）

Supplementary Figure 10. (a) The possible Pain-related mechanism diagram of CP/CPPS. Prostate tissue of CP/CPPS model rat produces a variety of inflammatory mediators (sensitizers), which act on homologous receptors expressed by nociceptors to activate the cAMP-PKA pathway and PLC-PKC pathway of intracellular signal transduction. These pathways can phosphorylate TRPV1, causing peripheral sensitization, converting chemical signals into electrical signals, then transmitting to the central system, and reducing the pain threshold. **(b)** the possible analgesic mechanism diagram of EA on CP/CPPS. EA on acupoints may interfere with the activation of the cAMP-PKA pathway and PLC-PKC pathway, hindering intracellular signal transduction, reducing the phosphorylation of TRPV1, then causing peripheral desensitization, increasing the pain threshold, and do not transmit noxious stimulation to the central system, to effectively for pain relief. "⟶"Directly acting on downstream substances,"--->" Indirect action on downstream substances.

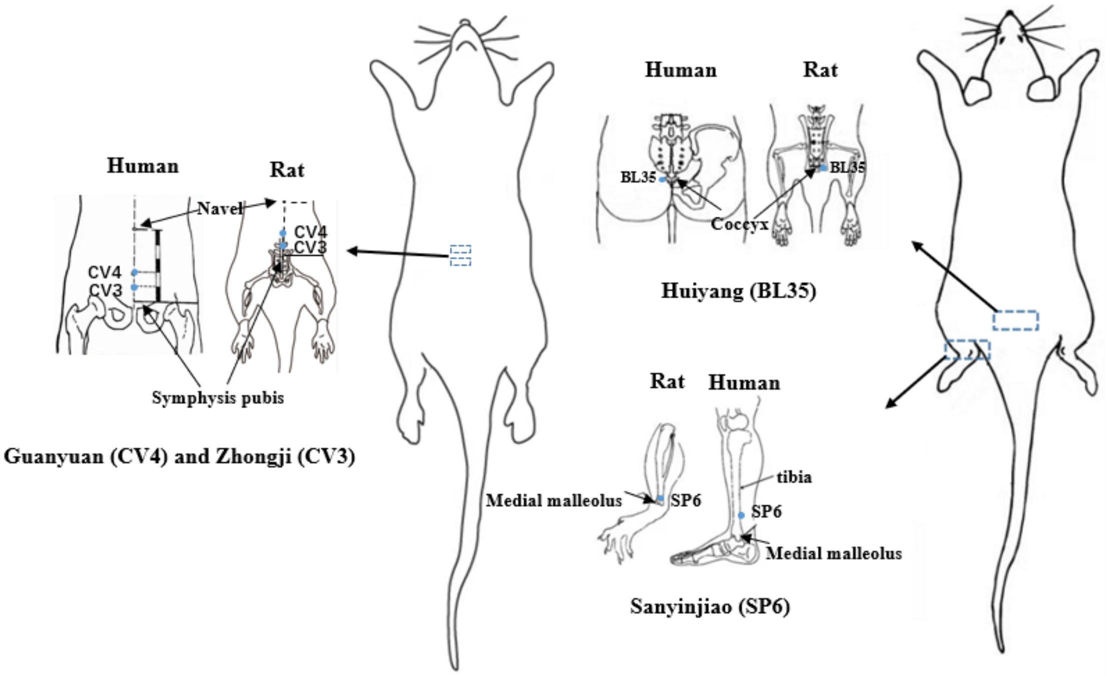

Supplementary Figure 11. Schematic representation of the points in the EA group and sham group. Anatomical localization of the acupoints are shown on the rat and human bodies. Points are indicated by blue dots.
