# Supplementary material for: Effect of electroacupuncture on cyclic adenosine monophosphate-protein kinase A-vanillic acid receptor subtype 1 of the transient receptor potential/PLK-protein kinase C-vanillic acid receptor subtype 1 of the transient receptor potential pathway based on RNA-seq analysis in prostate tissue in rats with chronic prostatitis/chronic pelvic pain syndrome
Source: Front Neurosci. 2022 Aug 24;16:938200. doi: 10.3389/fnins.2022.938200 (PMC9449126; doi:10.3389/fnins.2022.938200)
Supplement: Supplementary file 2 [file Table_1.docx]

| **Statistics of up-regulated genes and down-regulated genes of intersection genes between pmodel / sham and pmodel / EA** | | | | | | | |
| --- | --- | --- | --- | --- | --- | --- | --- |
| **Upregulation** | |  |  | **Downregulation** | |  |  |
| **No.** | **Gene ID** | **Gene name** | **P-value** | **No.** | **Gene ID** | Gene name | P-value |
| 1 | novel.384 | - | 0.012506986 | 1 | 108350169 | - | 0.028406577 |
| 2 | 108349500 | LOC108349500 | 0.005107681 | 2 | 298475 | LOC102550609 | 0.021842075 |
| 3 | 691695 | LOC691695 | 0.006161879 | 3 | 108350941 | LOC108351539 | 0.044231181 |
| 4 | 108350592 | LOC108350592 | 0.0219589 | 4 | 140662 | Agr2 | 0.009594511 |
| 5 | 289515 | Epgn | 0.003121427 | 5 | 688699 | - | 0.001002885 |
| 6 | 103690142 | LOC103690142 | 0.000713737 | 6 | novel.1701 | LOC100911725 | 0.025875427 |
| 7 | 366752108 | LOC366752 | 0.012803035 | 7 | 295695 | LOC108352776 | 0.042654331 |
| 8 | 108351885 | LOC108351885 | 0.00349009 | 8 | novel.999 | Mir664-2 | 0.04994514 |
| 9 | 108350774 | LOC108350774 | 0.004772638 | 9 | 64632 | - | 0.008405011 |
| 10 | 100911379 | LOC100911379 | 0.008572464 | 10 | novel.1058 | LOC102555981 | 0.011968979 |
| 11 | 102552182 | LOC102552182 | 0.007201843 | 11 | novel.1702 | LOC103689977 | 0.047104344 |
| 12 | 298643 | Miip | 0.00442471 | 12 | 103689977 | LOC108353138 | 0.008251096 |
| 13 | 102549213 | LOC102549213 | 0.018189511 | 13 | 108348407 | Slc9a2 | 0.006684762 |
| 14 | 102550354 | LOC102550354 | 0.025529843 | 14 | 503009 | Penk | 0.003317585 |
| 15 | 295322 | Vtcn1 | 0.002224734 | 15 | 103694416 | LOC310926 | 0.007126091 |
| 16 | 24334 | Eno2 | 0.009119974 | 16 | novel.1766 | Atp8a2 | 0.004293071 |
| 17 | 108348110 | LOC108348110 | 0.016595381 | 17 | 83834 | Rasd2 | 0.003886532 |
| 18 | novel.531 | - | 0.021282696 | 18 | 171099 | LOC102556275 | 0.031169773 |
| 19 | 312102 | Vsig4 | 0.000514995 | 19 | 295277 | - | 0.002969401 |
| 20 | 108352383 | LOC108352383 | 0.00185506 | 20 | 306956 | - | 0.041358332 |
| 21 | 108350525 | LOC108350525 | 0.041243654 | 21 | novel.234 | - | 0.01989929 |
| 22 | novel.103 | - | 0.043768903 | 22 | novel.1904 | Lgals4 | 0.021419355 |
| 23 | 103693683 | LOC103693683 | 0.001139692 | 23 | 307393 | RGD1564801 | 0.01645967 |
| 24 | 108349468 | LOC108349468 | 0.01499318 | 24 | novel.1330 | Tnfrsf19 | 0.044745393 |
| 25 | 103692128 | LOC103692128 | 0.01273095 | 25 | 25134 | Plekhd1 | 0.024078145 |
| 26 | 102548141 | LOC102548141 | 0.031894388 | 26 | novel.1414 | Rab6b | 0.025127673 |
| 27 | 100361292 | Fam205a | 0.002687206 | 27 | 363123 | - | 0.028906275 |
| 28 | 289250 | Olr1588 | 0.019100005 | 28 | novel.1377 | Dnmt3b-ps2 | 0.035804776 |
| 29 | 103690099 | LOC103690099 | 0.049160236 | 29 | 365901 | Hspb6 | 0.000561386 |
| 30 | 83610 | Apba2 | 0.015668559 | 30 | 362466 | - | 0.028154976 |
| 31 | 494209 | Ly49si3 | 0.004810464 | 31 | 294011 | Otub2 | 7.26E-05 |
| 32 | 102551724 | LOC102551724 | 0.004060775 | 32 | 503568 | LOC102548818 | 0.032322826 |
| 33 | 500349 | Gprc5d | 0.015903818 | 33 | 305475 | Nupr1 | 0.0147394 |
| 34 | 290729 | Neil3 | 0.006732548 | 34 | 64392 | LOC102549491 | 0.025338691 |
| 35 | 288272 | Mis18a | 0.005676369 | 35 | 102549491 | Ankrd35 | 0.028081163 |
| 36 | 499413 | Hsf2bp | 0.000633724 | 36 | novel.1416 | Cck | 0.02743871 |
| 37 | novel.1532 | - | 0.013701084 | 37 | 288321 | - | 0.021537078 |
| 38 | 297783 | Mybl1 | 0.006697501 |  |  |  |  |
| 39 | 102551116 | LOC102551116 | 1.82E-05 |  |  |  |  |
| 40 | 266603 | Aldh1a3 | 0.001194451 |  |  |  |  |
| 41 | novel.824 | - | 0.002199302 |  |  |  |  |
| 42 | 102547581 | LOC102547581 | 0.042856794 |  |  |  |  |
| 43 | novel.1907 | - | 0.008228595 |  |  |  |  |
| 44 | 685611 | Phldb2 | 0.00525476 |  |  |  |  |
| 45 | 288227 | Bace2 | 0.000819627 |  |  |  |  |
| 46 | 65146 | Pirb | 0.025460157 |  |  |  |  |
| 47 | 108349480 | LOC108349480 | 3.37E-05 |  |  |  |  |
| 48 | novel.1899 | - | 3.19E-05 |  |  |  |  |
| 49 | 102552371 | LOC102552371 | 0.030041358 |  |  |  |  |
| 50 | 681249 | Fam72a | 0.005369333 |  |  |  |  |
| 51 | 100911432 | LOC100911432 | 0.015592771 |  |  |  |  |
| 52 | 100911825 | LOC100911825 | 0.01199481 |  |  |  |  |
| 53 | 100361383 | Ecm2 | 0.00083428 |  |  |  |  |
| 54 | novel.1930 | - | 0.006254745 |  |  |  |  |
| 55 | 308569 | Acpt | 0.042991738 |  |  |  |  |
| 56 | 100909879 | LOC100909879 | 0.009369235 |  |  |  |  |
| 57 | novel.1216 | - | 0.009723767 |  |  |  |  |
| 58 | 501869 | Sh2d1b | 9.06E-06 |  |  |  |  |
| 59 | 108350704 | LOC108350704 | 0.049023771 |  |  |  |  |
| 60 | 100910286 | LOC100910286 | 0.035262735 |  |  |  |  |
| 61 | 407757 | Krt76 | 0.023364203 |  |  |  |  |
| 62 | 689756 | Tnip3 | 0.01095848 |  |  |  |  |
| 63 | 64037 | Slc22a4 | 0.016219979 |  |  |  |  |
| 64 | 65053 | Rassf9 | 0.002199827 |  |  |  |  |
| 65 | novel.1044 | - | 0.00100455 |  |  |  |  |
| 66 | 684555 | Gp6 | 0.001933771 |  |  |  |  |
| 67 | 291179 | Gpr141 | 0.006681914 |  |  |  |  |
| 68 | 116560 | Kcnj9 | 0.039106452 |  |  |  |  |
| 69 | 100909750 | LOC100909750 | 0.029823639 |  |  |  |  |
| 70 | 414783 | RT1-CE4 | 0.008909294 |  |  |  |  |
| 71 | 289337 | Tlr5 | 0.031377255 |  |  |  |  |
| 72 | 316326 | Neurl3 | 0.037447943 |  |  |  |  |
| 73 | 501738 | Cd300lb | 0.031591699 |  |  |  |  |
| 74 | 266714 | Myo7a | 0.000348248 |  |  |  |  |
| 75 | 305841 | Olr1637 | 0.031655662 |  |  |  |  |
| 76 | 192253 | Myo16 | 0.001497929 |  |  |  |  |
| 77 | 361734 | Ms4a4a | 0.010544343 |  |  |  |  |
| 78 | 83810 | Trpv1 | 0.048541033 |  |  |  |  |
| 79 | 362848 | Pram1 | 0.001693419 |  |  |  |  |
| 80 | 24906 | LOC24906 | 0.017173119 |  |  |  |  |
| 81 | 29403 | Asgr2 | 0.005078636 |  |  |  |  |
| 82 | 25383 | Apobec1 | 5.74E-05 |  |  |  |  |
| 83 | 298077 | RGD1305807 | 0.031231734 |  |  |  |  |
| 84 | 25116 | Hsd11b1 | 0.000860315 |  |  |  |  |
| 85 | 100360801 | Ddx60 | 0.006771918 |  |  |  |  |
| 86 | 681325 | LOC681325 | 0.001960037 |  |  |  |  |
| 87 | 286921 | Akr1b8 | 0.027364731 |  |  |  |  |
| 88 | 368153 | RT1-CE7 | 0.029469031 |  |  |  |  |
| 89 | 298534 | Clspn | 0.034728605 |  |  |  |  |
| 90 | 502640 | Cerkl | 0.010989228 |  |  |  |  |
| 91 | 100911030 | LOC100911030 | 0.017350184 |  |  |  |  |
| 92 | 103693438 | LOC103693438 | 0.0450658 |  |  |  |  |
| 93 | novel.690 | - | 0.006485748 |  |  |  |  |
| 94 | 690097 | LOC690097 | 0.000172243 |  |  |  |  |
| 95 | 292594 | Lilrb4 | 0.047133226 |  |  |  |  |
| 96 | 108353247 | LOC108353247 | 0.014124611 |  |  |  |  |
| 97 | 83620 | Cit | 0.021241986 |  |  |  |  |
| 98 | 100362785 | Ankdd1a | 0.038054129 |  |  |  |  |
| 99 | 24891 | Abcb4 | 0.015844754 |  |  |  |  |
| 100 | 497942 | Cxcl16 | 0.014847555 |  |  |  |  |
| 101 | 298765 | Zfp36l2 | 0.01904239 |  |  |  |  |
| 102 | 102551940 | LOC102551940 | 0.010542915 |  |  |  |  |
| 103 | 100911190 | LOC100911190 | 0.021459049 |  |  |  |  |
